# School Performance among Children and Adolescents during COVID-19 Pandemic: A Systematic Review

**DOI:** 10.3390/children8121134

**Published:** 2021-12-04

**Authors:** Eleni Panagouli, Androniki Stavridou, Christina Savvidi, Anastasia Kourti, Theodora Psaltopoulou, Theodoros N. Sergentanis, Artemis Tsitsika

**Affiliations:** 1 2nd Department of Pediatrics, “P. & A. Kyriakou” Children’s Hospital, School of Medicine, National and Kapodistrian University of Athens, 11527 Athens, Greece; elenpana@med.uoa.gr (E.P.); stavroniki@hotmail.com (A.S.); chrisavvidi@gmail.com (C.S.); anastasiakourti.ak@gmail.com (A.K.); tsergentanis@yahoo.gr (T.N.S.); 2 Department of Clinical Therapeutics, “Alexandra” Hospital, School of Medicine, National and Kapodistrian University of Athens, 11528 Athens, Greece; tpsaltop@med.uoa.gr

**Keywords:** academic performance, COVID-19 pandemic, students, school closure

## Abstract

As a result of COVID-19 restrictions, conventional ways of schooling were not possible, and in order to continue the educational process new digital resources, such as online learning platforms, were imposed. Although virtual courses provided high-quality educational material, the efficiency in children’s and adolescents’ academic performance in general is yet to be known. The purpose of this systematic review is to examine whether the academic performance of school-aged students was impacted or not through online learning and modified educational methods during the ongoing COVID-19 pandemic. According to the studies, either students suffered from learning losses comparing to pre-pandemic years or, in some cases, they benefited from online learning, especially in mathematics. Younger students and students with neurodevelopmental disorders or special education needs seemed to suffer more. Parents/caregivers reported that their children’s performance deteriorated, while others thought that online learning was beneficial. Teachers also reported that students presented academic gaps and difficulties in mathematics and reading compared to typical years. Consequently, the new ways of schooling imposed by the restrictions have not been fully tested and the impact cannot be described thoroughly. The investment in technological equipment in schools for the majority of students, along with the training of teachers in digital competence, should be a priority.

## 1. Introduction

Since the spring of 2020 after the first outbreak of the SARS-CoV-2 virus, the COVID-19 pandemic has caused and continues to cause serious disturbances in daily life around the world [1]. To counteract this public health emergency, a number of measures were imposed globally, including home-confinement, social distancing, and school closures [2]. Among restrictive measures, school closure has changed children’s and adolescents’ everyday life, affecting their emotional resilience and mental health overall [3]. Pioneering this decision was China, where a nationwide school closure was imposed during the prolonged home-confinement period [4].

Due to restrictions, conventional ways of schooling were not possible and new ways of schooling had to be proposed in order to maintain an everyday routine and not disrupt the educational process. Homeschooling was a preferable means adopted by various educational settings and countries around the world [5]. As a pedagogical method, homeschooling has been well established since the 1970s in some countries such as the US, where parents, relatives, or tutors provided basic education at home [6]. During that time, concerns were raised regarding students’ assessments, school performance, and socialization, as well as educational materials for students with special educational needs [6]. Students need to feel that they act and learn in a safe environment where they are given instructions about which learning strategies to follow or consultation about interpersonal relationships that blossom in the school context [7].

During the ongoing pandemic, the homeschooling approach seemed to differ from one country to another, using mainly virtual courses delivered through digital resources such as online learning platforms [8]. A number of online learning resources were made available, including recorded classes, tutorials, online conferences, and educational platforms that offered online live interaction with teachers [9]. Teachers played a significant role in providing educational materials and assessing students’ performance through online exams and homework activities [8,10]. Homeschooling and online learning as altered learning styles were used for the first time in such a global context as emergency remote education, raising concerns about their efficiency. Inadequacies in education opportunities were highlighted during the ongoing pandemic, where the socioeconomic status of parents could not always provide the necessary means for online learning or the professional help children may need [5,11]. Children with special educational needs or neurodevelopmental disorders were at greater risk of presenting educational gaps due to lack of face-to-face interaction with the teachers [12,13,14,15,16,17,18,19]. Moreover, the uncertain educational future impacted students’ academic achievement, which can be enforced by a stressful life event, such as a global pandemic [20].

Although online learning was an ideal setting through the lockdown period, its efficiency in children’s and adolescents’ academic performance in general is yet to be known. The purpose of this systematic review is to examine whether the academic performance of school-aged students was impacted or not through online learning and modified educational methods during the ongoing COVID-19 pandemic.

## 2. Materials and Methods

### 2.1. Study Design

A recommended reference framework for systematic reviews was performed, following the PRISMA guidelines for systematic reviews. The research was conducted in the following databases: PubMed, Google Scholar, ERIC, SCOPUS, DOAJ, and PsycNet, up to 18 July 2021. The algorithm used was the following: (COVID-19 OR SARS-CoV-19 OR SARS-CoV-2 OR “2019-nCoV” OR “novel coronavirus”) AND (“school performance” OR “academic performance” OR learning OR e-learning OR “distance learning” OR “school closure” OR e-classes OR “school grades” OR “academic grades” OR “school notes” OR “academic notes” OR “school attainment” OR “academic attainment” OR “educational attainment”) AND (child OR children OR kid OR kids OR youngster OR youngsters OR adolescent OR adolescents OR teen OR teens OR teenager OR teenagers). References of eligible studies and relevant reviews were searched using a snowballing technique.

### 2.2. Inclusion Criteria

All articles were considered eligible for this research if they examined school performance in school-aged children and adolescents during the COVID-19 pandemic based on evidence from student reports or students themselves. Kindergarten children were excluded. Additionally, due to homeschooling, as opposed to restrictive measures, studies involving parents’ observations were also collected in order to provide more data that were plausible. Furthermore, as teachers are the main evaluators of children’s and adolescents’ performance, their observations were valuable and were also collected. Regarding study design, only case reports, cohort studies, cross-sectional studies, case series, and case-control studies were selected. There were no language restrictions. Two authors (A.S. and C.S.) worked independently in pairs to perform the selection of studies.

### 2.3. Data Extraction and Analysis

Three reviewers (A.S., C.S., and E.P.) reviewed the articles simultaneously and independently through a piloted data extraction form. The following variables were used to extract data for each study: name and identity of the article, name of first author and year of publication, region/country where the survey was conducted, language, study period, study design, sample size, age range, and selection of sample, ascertainment and/or association with COVID-19 pandemic, outcomes, statistical analysis, and main findings. Any disagreement was resolved through reviewer discussion and team consensus.

### 2.4. Quality Assessment

The quality assessment was performed by two independent reviewers (A.S. and C.S.) during the screening process, evaluating the risk of bias in eligible studies through the Newcastle-Ottawa Scale for cross-sectional studies and cohort studies accordingly.

## 3. Results

### 3.1. Selection of Studies

**Figure 1:**
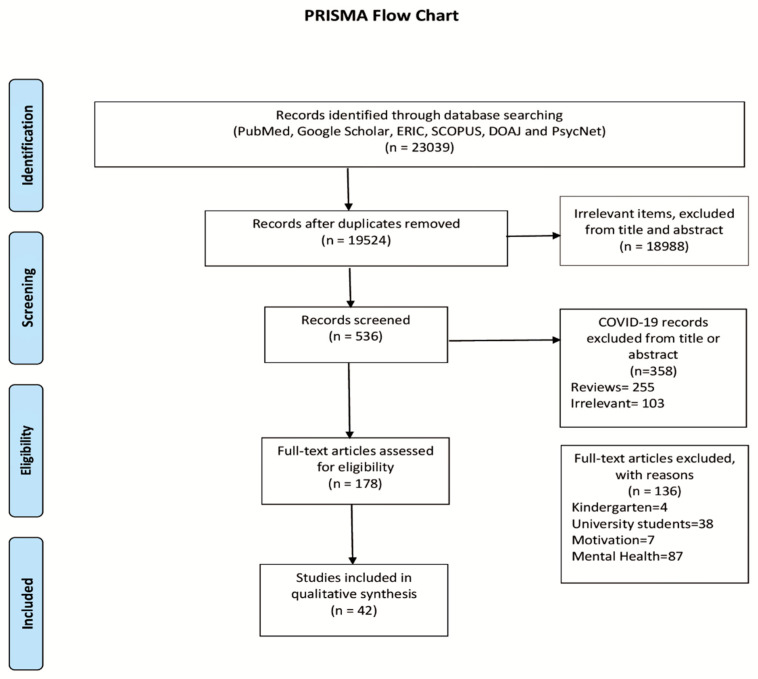
PRISMA flow chart.

The research in databases retrieved 23,039 publications, from which 3515 were duplicates. After screening a total of 19,524 records by title, abstract, and full text, 42 of them were finally considered eligible [7,8,10,11,12,13,14,15,16,17,18,19,21,22,23,24,25,26,27,28,29,30,31,32,33,34,35,36,37,38,39,40,41,42,43,44,45,46,47,48,49,50] (Prisma Flow Chart—Figure 1).

The demographic characteristics are summarized in Table 1. The majority of the studies were conducted in Europe (n = 19 studies) [13,17,18,19,21,22,23,24,25,26,27,28,29,30,31,32,33,34,35], followed by Asia (n = 13 studies) [7,8,10,11,15,16,36,37,38,39,40,41] and America (n = 7 studies) [12,14,42,43,44,45,46,47], while two studies were conducted in Africa [48,49] and one in Oceania [50]. The performance of 15,385,942 students was assessed. Furthermore, 143,511 teachers and 22,203 parents participated in 19 studies in order to report their students’ and children’s performance, correspondingly. It should be pointed out that in one study the participants were 15 teachers and parents (half of them had double roles), without discriminating the exact number of teachers or parents [16]. Moreover, in one study 5832 schools participated without mentioning the total number of students [35]. Although the majority of the studies referred to students, the age range in most of them was unclear. In some studies where the subjects’ age was defined, the age range of the students was between 8 and 22 years old. Concerning the study design, 17 of the studies were cross-sectional [7,8,12,14,15,17,18,25,26,28,31,32,33,34,36,40,41] and 13 were cohort studies [19,21,23,24,27,29,30,37,38,45,46,49,50], while the rest were case studies (n = 5) [10,13,16,42,48] or mixed design studies (n = 5) [11,22,35,44,47]. One study was a qualitative description study [39] and one was a qualitative projection study [43] (Table S1).

### 3.2. Students

The new way of online learning as imposed by the school closure affected all aspects of students’ life. According to the studies, students either suffered from learning losses compared to pre-pandemic years [23,24,29,34,38,45,46,49,50] or, in some cases, they benefited from online learning [10,22,27,30,33,34,37,39,40,43]. More specifically, greater emphasis was given concerning academic performance in two sections, mathematics [10,29,34,43,46,50] and reading [29,43,46], with mixed results. Most of the students presented a significant decrease in mathematics scores compared to previous years [34,43,46,50], while a small number maintained their math performance [10] or even increased it, especially low-achieving students [26,29]. On the other hand, concerning mostly high-achieving students, they either maintained or gained reading skills [29,43,46]. Low-achievement students, who either presented improvements [7,30,37] or performed poorly, acquired greater changes in performance [25,34]. Younger students faced more difficulties during online learning [23,33], however they presented more enthusiasm for learning materials because they were more creative and interactive [22]. Overall, 34% of younger and 21% of older pupils reported not learning many new things due to tasks being repetitive and simple [22].

### 3.3. Parents/Caregivers

During the lockdown, parents and caregivers played an important role in children’s education because classes were implemented online and mostly at home. Thus, parents or caregivers could monitor their children’s performance and voice concerns. Some of them reported that their children’s performance deteriorated [11,21,28,31,42], while others thought that online learning was beneficial due to unaffected contact with the teachers [32,36,41]. Parents or caregivers believed that younger children could not discipline or self-regulate in order to attend online classes and do their homework [11] while parental support was present [22,33]. Many of them were concerned because they could not always provide the help needed by their children due to lack of experience with new technologies [11]. Socioeconomic inequalities played a significant role in access to online learning, affecting the performance of students of all grades, especially in low-income communities [24,26,46,49,50].

### 3.4. Teachers

The main role in evaluating children’s performance was occupied by teachers in all grades. The COVID-19 restrictions prohibited face-to-face interaction with the students, entering a new method of online learning. According to teachers, students presented academic gaps and difficulties in mathematics and reading compared to typical years [14,15]. The lack of in-person consultation, especially in science subjects, was believed to be an aggravating factor for students’ performance [48]. A significant number of students did not participate in online classes, mostly from non-privileged areas, increasing the drop out risk, as reported by teachers [15,45]. In addition, teachers raised concerns for students with special disabilities and non-native speakers [44]. The interactive and interesting material used in online classes increased the interest of younger students, while good communication between teachers and parents/caregivers could provide learning activities that were better in quality [8,10].

### 3.5. Students with Neurodevelopmental Disorders or Special Education Needs

Although life for all students through the COVID-19 pandemic created many challenges, students with neurodevelopmental disorders or special education needs struggled the most. Children and adolescents with ADHD faced difficulties in concentrating in online classes and completing learning activities due mainly to inattention [12,13]. Many of them struggled with online learning and they were at high risk for dropping out or depression [47]. Similarly, students with special education needs and disabilities appeared to have difficulties, not only academically, but also socially, increasing the drop out risk and the time spent in front of screens [14,15,16,17,18,19]. Specifically, students with dyslexia did not reach their reading goals and presented difficulties in reading, comprehension, and mathematics during online classes [18,19]. As reported by their parents, students with dyslexia were less motivated in establishing reading routines and were negatively affected during the quarantine [19].

### 3.6. Risk of Bias

The Newcastle-Ottawa Scale was used for assessing cross-sectional and cohort studies. Qualitative studies were not assessed. Regarding the cross-sectional studies, all of them scored between four and eight points in the Newcastle-Ottawa Scale, while the majority of cohort studies were assessed as good quality studies. In many cases the representative of the sample was mainly students, although the sample size was satisfactory. The tool used to assess the academic performance (online questionnaires, interviews, observations, exam results, software) was described in detail, but in many cases was not validated. The control of confounding factors was performed under statistical analysis, with the outcome being assessed by self-reporting or record linkage.

## 4. Discussion

Online learning created a new educational reality, which either benefited students or promoted educational loss. According to the present systematic review, the majority of studies referred to educational losses during the ongoing COVID-19 pandemic when compared to the pre-pandemic era [23,24,29,34,38,45,46,49,50]. Similarly, studies predicted that students during the COVID-19 pandemic may face “learning losses”, accompanied with challenges in mental health and well-being [10,50,51,52]. On the other hand, according to our findings, there were also a number of students who benefited from online learning [10,22,27,30,33,34,37,39,40,43]. This could be due to the short period of lockdowns in some countries and the lack of substantial time to evaluate their academic performance [50].

According to the present systematic review, eligible studies, which evaluated performance in mathematics and reading skills during the ongoing COVID-19 pandemic, provided mixed results. Some students increased their performance [26,29] while others presented low scores [34,43,46,50]. Specifically, differences occurred among younger students, who seemed to struggle more during online classes compared to older ones [23,33], but who presented more enthusiasm due to more interactive and creative materials [22]. Following these findings, older students seem to be more autonomous learners and familiar with technology. Thus, online learning was not an issue compared to the younger ones [53]. The absence of face-to-face interaction with teachers may lead to biased conclusions about academic performance in general, due to lack of feedback with the students [53].

According to our findings, economic inequalities played a significant role in academic performance due to lack of necessary means, such as laptops/tablets and internet connection issues, in order for students to attend online classes [24,26,46,49,50]. The ongoing COVID-19 pandemic highlighted this issue, as was stated in various articles [7,54,55]. Parents and caregivers were the main providers, and, in many cases, they failed to help their children due to lack of education and familiarization with technology [11,22,33]. On the other hand, according to our findings, parents and caregivers spent more time with their offspring as they all stayed home and could easily monitor their school behavior and performance [11,21,28,31,32,36,41,42].

Distance learning was used as an emergency measure during the COVID-19 pandemic [56]. Thus, there were cases where both students and teachers faced difficulties in accessing electronical devices and/or an internet connection, while there were also students who did not have a quiet place to study, especially in disadvantaged families. Many teachers, on the other hand, did not have the technical and pedagogical skills needed to integrate digital devices in instruction, and consecutively in distance education [57]. Even skilled teachers experienced difficulties in adapting to distance learning demands [56], not only in terms of teaching but also in offering psychological and communication proximity to their students, as pedagogy requires [58].

The role of teachers includes the evaluation of students’ performance during the academic year, among other responsibilities. The use of online learning created a need for new ways of assessment to emerge. According to the present systematic review, teachers observed a deterioration in students’ performance, mostly in mathematics and reading [14,15]. Communication problems, limited attendance to classes, and failure in monitoring students were some of the problems teachers had to face [15,45,49]. Concerns about the consequences of limited communication between teachers and students were raised, especially for younger students who need more support [53]. Furthermore, the group of students most affected by the ongoing COVID-19 pandemic was those who presented neurodevelopmental disorders or special education needs. According to our findings, students with ADHD could not concentrate due to inattention [12,13] and those with dyslexia could not maintain their reading goals [18,19]. In many cases, the drop out risk increased due to lack of socialization and achievement failures [15].

It is worth mentioning, however, that distance learning (and information and communication technology, in general) may benefit students with learning disabilities, providing them an environment where they can gain motivation, engagement, and interest, and which benefits their attention and the cooperation between students [59].

Although this is not the first systematic review that examined the performance of students during the COVID-19 pandemic [60], we researched a variety of factors that contributed to the evaluation of academic performance. Self-report evaluation by the students, in accordance with teachers’ and parents’/caregivers’ opinions, presented a more accurate picture. The evaluation of school performance should provide information about the learning losses of students throughout the year, focusing on the subjects that should be reinforced. In this case, new study programs should be established to help students accomplish their academic goals.

Concerning limitations, the fact that many studies chose to examine mathematics and reading performance may have led to biased results due to discrimination in subject selection. Furthermore, the use of specific programs could create a routine, making it easier for students to be more effective, therefore the results may not be accurate. Due to home confinement, parents monitored their children’s and adolescents’ performance, and they expressed their opinions, which were influenced by their stress levels and socioeconomic status [61]. Especially for students with neurodevelopmental disorders or special education needs, parents were more stressed and cautious because access to teachers and health professionals was limited, thus causing parents to overanalyze their children’s performance.

## 5. Conclusions

The present systematic review highlights in depth the consequences of the school closures during the pandemic. Academic performance of students of all grades seemed to have been negatively influenced, while younger students and students with neurodevelopmental disorders or special education needs were reported to have suffered more. We considered it important that these results were recorded not only by students but by parents, caregivers, and teachers as well. According to our findings, academic gaps and difficulties in lessons such as mathematics and reading were reported, which should be taken into consideration. It is important to design the following years in the educational systems in order to fill those gaps. On the other hand, in some cases students benefited from online learning, and those positive effects should also be taken into consideration. The investment in technological equipment in schools in order to provide for the majority of students, along with the training of teachers in digital competence, should be a priority.

Nevertheless, the new ways of schooling imposed by the restrictions have not been fully tested and the impact cannot be described thoroughly. The COVID-19 pandemic set a prerequisite for the use of alternative methods of schooling that could be useful, not only in times of crisis, but also in helping students with serious illnesses and special needs. A program that could focus on a student’s emotional needs and well-being should be established as part of reintegration, in cooperation with teachers and parents/caregivers. Special focus should be given to younger ages, which seemed to struggle the most.

## Figures and Tables

**Table 1:** Description of studies examining school performance in children and adolescents.

First Author (Year)	Region, Country	Selection of Sample	Main Findings
Amelia et al. (2020) [10]	Cimahi, Indonesia	Teachers (4) and students (6) from junior high school in Cimahi	According to results, five out of six students scored above MCC (minimum completeness criteria), showing that e-learning did not disturb the learning of mathematics. Achievement and learning activities could be increased through good communication between teachers and students.
Andrew et al. (2020) [21]	UK	Parents of children aged 4–15	There was a decrease in hours engaged with learning activities in children, from 90% before lockdown to 60% during lockdown. Secondary school students presented the bigger decrease from 6.59 to 4.15 h. The majority of them spent around 2 to 4 h in learning activities, while a substantial minority (20%) spent less than 2 h.
Bansal et al. (2020) [36]	India	Parents with children using remote learning	Responders believed that remote learning was beneficial because it was safe (89.9%), helped with keeping in touch with teachers and classmates (61.6%), and with learning material (63.1%).
Becker et al. (2020) [12]	US	Adolescents (118 with ADHD) from the 9th through 11th grades and their parents	During the COVID-19 pandemic, 72% of the adolescents spent 3 h or less on schoolwork on an average school day. Specifically, more difficulties and fewer routines were reported from adolescents with ADHD than those without. Adolescents with ADHD experienced more difficulty in concentrating and remote learning, higher negative affect, and fewer adolescent routines due to COVID-19.
Bobo et al. (2020) [13]	France	Parents of children and adolescents with ADHD	Children with ADHD struggled to complete school-related tasks as reported by their parents, with the role of inattention being one of the main factors in children’s learning difficulties.
Bubb and Jones (2020) [22]	Norway	Parents, teachers, and pupils (6–16 years)	The youngest pupils showed more enthusiasm in more creative tasks assigned by teachers during homeschooling. In total, 79% of younger pupils agreed that they had learned many new things, while 65% of older students agreed. However, a significant percentage of pupils (34% of younger students and 21% of older) reported not learning many new things because the tasks were repetitive and simple. In addition, 62% of older pupils stated that they had more homework, and they were more concentrated at home. Parental support enhanced pupils work at home and their independence concerning their tasks.
Dong et al. (2020) [11]	China	Parents through Wenjuanxing platform	The parents generally had negative beliefs regarding online learning and preferred learning in school, at least for younger ages. They believed that their children could not self-regulate and lacked discipline in doing their homework. Furthermore, the lack of professional knowledge in supporting their children during online learning was considered an aggravating factor. During the COVID-19 pandemic, only a small percentage of parents stated that online learning had better learning outcomes (11.0%) and generated improvement in several skills including language development (21.2%), literacy (25.2%), social skills (24.8%), independent skills (17.8%), arts (21.1%), and physical health (10.9%).
Goodrich et al. (2020) [14]	US	Elementary school teachers	According to the teachers, students presented larger academic gaps in the fall of 2020 when compared to typical years, with many of them not ready for transitioning to the next grade. Moreover, 73.9% and 69.7% of teachers indicated difficulties in reading and mathematics, respectively, which was larger than typical years. Similar findings reported by teachers for students with disabilities, where 58.5% of teachers confirmed achievement gaps larger than typical years.
Kuhfeld et al. (2020) [43]	US	Seventh-grade students who took the MAP Growth assessments in 2017–2018	Students attending school in fall 2020 were estimated to return with approximately 70% reading gains relative to a typical school year. However, in mathematics students’ estimation was smaller, fluctuating from 37% to 50% compared to a typical school year. Although those projections may not be universal, the majority of students were making gains in reading.
Maldonado and De Witte (2020) [35]	Belgium	Schools in Flanders	In 2020, students experienced significant learning losses in all subjects, while mathematics scores presented the biggest decrease in school averages (SD = 0.19) and Dutch scores (SD = 0.29) compared to previous years.
Moghli and Shuayb (2020) [15]	Jordan, Lebanon, and the Occupied West Bank and Gaza Strip	Teachers (274), students (105), and parents (299)	According to teachers, a significant rate of dropout was noted in non-formal schools, with 77% of the students not participating in distance learning. The majority of them were boys (70%), with more than half (53%) being children with special educational needs. In addition, 15% of students reported a decline in their academic performance.
Putri et al. (2020) [16]	Indonesia	Teachers and parents of two primary schools in Tangerang, Indonesia.	Some of the challenges faced by students included the limitation of social contact and communication, and difficulties appeared in students with special educational needs and increased time in front of screens.
Sintema (2020) [48]	Zambia	Teachers at a public secondary school in Chipata District of Eastern Province in Zambia	According to the teachers’ views, the possibility of a decline in performance of secondary school students in national examination was likely to occur. The reduction of face-to-face interaction with the teachers was considered an aggravating factor due to lack of consultation from the teachers, especially in science subjects.
Tomasik et al. (2020) [23]	Switzerland	Pupils from MINDSTEPS system (grade 3 to 9)	In terms of learning gains, students in secondary school remained unaffected, while primary school students presented a decrease in learning.
Zhang Qing et al. (2020) [7]	China	Middle school students	There was a positive correlation between emotional and learning management skills (r = 0.498, *p* < 0.01). Learning management skills were predicted by positive emotional ability, while low- and high-scoring groups presented significant differences in learning management skills (t = –14.69, *p* < 0.001).
Zhao Ying et al. (2020) [8]	China	Students (738), parents (1062), and teachers (210)	According to teachers, online learning increased students’ interest, mostly in younger students. Furthermore, half of the teachers expressed that homeschooling would have a negative impact on academic performance of their students. Schooling in classrooms was considered a more preferable way of learning, as stated by both parents and teachers.
Banerjee et al. (2021) [17]	UK	Parents of children and young people (CYP) with special educational needs and disabilities (SEND)	A significant number of parents (n = 18) reported that their children and young people with special educational needs and disabilities were affected emotionally and academically by the COVID-19 pandemic.
Baschenis et al. (2021) [18]	Italy	Students with and without dyslexia	Children with dyslexia presented more difficulties in online classes and in reading, comprehension, and mathematics, as confirmed by their parents. More than half (59 to 63%) did not reach the expected goals of reading skills.
Catalano et al. (2021) [44]	NY, US	K-12 teachers from New York State, largely from Long Island and New York City	According to teachers, students failed to regularly complete their assignments (nearly 30%), with this being seen mostly in students from less privileged areas. Concerns were raised for educational outcomes in students with disabilities (SWDs) and English language learners (ELLs).
Clark et al. (2021) [37]	China	Students who were in the last semester of their middle school education	During lockdown, student academic results improved by 0.22 SD through online learning, especially for those students who received online lessons from experts rather than their own teachers. Students using computers presented better performance than those using smartphones for online classes. Although students with high performance maintained their performance throughout lockdown, those with low performance benefited more.
Cui et al. (2021) [38]	China	Parent-child pairs of elementary school	The majority of students performed poorly in online classes and their performance decrease was statistically significant as time lapsed (*p* = 0.047). Greater performance in online classes was presented by students in grade 1 (23/46, 50%) and in grade 6 (13/31, 41.9%).
Engzell et al. (2021) [24]	Netherlands	Students in grades 4–7	A learning loss equal to 0.08 SD was revealed according to the results. That loss was higher in students with parents with low education levels (60%), enhancing the inequality among families and children during the pandemic.
Gore et al. (2021) [50]	Australia	Students	According to the results, Year 3 students coming from less advantaged schools presented lower achievement growth in mathematics. There were no significant differences among indigenous students and students from regional locations.
Hernawati et al. (2021) [39]	Indonesia	Students from sixth grade in elementary school Kamarung 1	Students’ learning outcomes increased after using videos as a learning technique, as opposed by the post-test and pre-test score evaluations.
Lichand et al. (2021) [45]	Brazil	Observations for middle and high school students	The distance learning increased dropout risk by 365%, with the enhancing number of COVID-19 cases in the area promoting school closure. Students learned only 27.5% of the study material, while the average standardized test scores presented a decrease of 0.32 SD. Remote learning affected students’ learning more than the impact of COVID-19 infection cases. The re-opening of schools increased the test scores of high school students by 20%.
Ma et al. (2021) [40]	China	Parents of children aged 7–15	Most of the parents (44.3%) reported that online learning was effective in gaining knowledge and improving skills, not only for practical skills but also in communication.
Mælan et al. (2021) [25]	Norway	Students in eighth to tenth grade	Students with low achievements tended to present lower efforts and self-efficacy, making it difficult for them to follow the class curriculum when schools reopened.
Meeter (2021) [26]	Netherlands	Pupils in grades 2 through 6	At the end of the year, students presented higher scores in mathematics than the previous year, with weaker students and students from less fortunate populations presenting the highest scores.
Patarapichayatham et al. (2021) [46]	US	Students from pre-kindergarten to grade 6	School closure due to COVID-19 pandemic caused greater learning losses in mathematics than reading, with results varying throughout the grades. According to the school status, in low poverty schools students presented more learning losses than in high poverty schools.
Poulain et al. (2021) [27]	Germany	Children	Children spent significantly more time doing schoolwork when receiving online learning materials regularly than those who received them irregularly.
Sabates et al. (2021) [49]	Ghana, Malawi	Children	During the transition period, a learning loss of 66% was estimated. That estimation created wider gaps in learning losses due to lack of home support and economic resources.
Scarpellini et al. (2021) [28]	Italy	Mothers of primary and middle school students	In primary school, the majority of students completed the homework (90.5%) which consisted mainly of revisions (74.3%) and no grade attribution (43.8%), with 11.5% of students not receiving any grades. On the contrary, in middle school students’ tests were planned (77.7%) and their grades varied from previous performance, with lower grades being almost twice as likely in students in primary school (OR = 0.49, CI 0.30–0.78).
Schult et al. (2021) [29]	Germany	Fifth graders	Compared to previous years, the competence of incoming fifth graders in 2020 was on average lower (−0.07 SDs for reading comprehension, –0.09 SDs for operations, and –0.03 SDs for numbers). High-achieving students presented greater differences in reading comprehension, with lower achievements in mathematics.
Sibley et al. (2021) [47]	US	Adolescents and young adults (A/YAs) with ADHD	During the first month of the pandemic, A/YAs with ADHD reported that social isolation (41.5%), boredom (21.3%), and difficulties in online learning (20.2%) were risk factors for depression and dropping out. On the contrary, more unstructured time to relax (39.4%), spending more time with family (29.8%), and more time available to complete academic work (21.3%) were marked as beneficial. A/YAs with higher IQs struggled more during the COVID-19 pandemic.
Soriano-Ferrer et al. (2021) [19]	Spain	Children with dyslexia and their mothers	Children and adolescents with dyslexia showed less reading activity and motivation during quarantine. The majority of parents reported that their children presented difficulties in establishing reading routines and were negatively affected due to the quarantine.
Spitzer and Musslick (2021) [30]	Germany	Students from grades 4 to 10	In 2020, during the school closure the performance of students increased compared to the former year. Low-achieving students presented greater improvements in performance.
Siachpazidou et al. (2021) [31]	Greece	Parents of children aged 4 to 12	School closure due to COVID-19 restrictions was believed by parents (48%) to be damaging for academic performance of their children.
Steinmayr et al. (2021) [32]	Germany	Parents	The frequency of student–teacher communication was associated with all academic outcomes in both samples. An exception was seen in elementary school. Distant teaching activities related to different academic outcomes of children in elementary school and secondary school.
Tus (2021) [41]	Philippines	Parents of junior high school students in private schools	The mean score of academic performance was satisfactory, revealing that students performed well during online classes.
Vainikainen et al. (2021) [33]	Finland	Pupils in grades 4 to 10 (N = 61,974) and parents of children in grades 1 to 10 (N = 39,186) through the Qualtrics survey system	According to pupils and parents, learning outcomes during distance learning did not vary among schools, with distance learning being less structured in primary grades and younger pupils requiring more support in the learning process.
van der Velde et al. (2020) [34]	Netherlands	Secondary education students	During lockdown, students from the highest educational scale were ahead of schedule more than students for lower scales compared to previous years. Students being more focused at home resulted in more accurate answers in study trials.
Yayci et al. (2021) [42]	Turkey	Parents of elementary school children	Half of the students had less than 60 min of average academic activity time per day, and only two of the students did not have any academic activity.

## Data Availability

Data are contained within the article.

## Supplementary Materials

The following are available online at https://www.mdpi.com/article/10.3390/children8121134/s1, Table S1: Further description of studies examining school performance in children and adolescents.
