# Supplementary material for: School Performance among Children and Adolescents during COVID-19 Pandemic: A Systematic Review
Source: Children (Basel). 2021 Dec 4;8(12):1134. doi: 10.3390/children8121134 (PMC8700572; doi:10.3390/children8121134)
Supplement: Supplementary file 1 [file children-08-01134-s001.zip › children-1482224-supplementary.pdf]

Table S1: Further description of studies examining school performance in children and adolescents.

First author (year)	Study period	Study design	Sample size	Age range	Ascertainment and/or association with the COVID-19 pandemic	Outcomes, way/questionnaires they were measured	Statistical analysis
Amelia et al. (2020) (10)	NR	Qualitative- case study	10	NR	COVID-19 pandemic	Questionnaires, interviews and observations	WhatsApp calls
Andrew et al. (2020) (21)	29 April-20 June 2020	Cohort	5582	NR	Lockdown	Online time diary of children activities, interviews, 2014–15 UK Time Use Survey (UKTUS) as a diary-based time use survey	Simple two-period regression model
Bansal et al. (2020) (36)	17-31 July 2020	Cross-sectional	2694	NR	COVID-19 pandemic	Online questionnaire	Descriptive statistics
Becker et al. (2020) (12)	16 May- 15 June 2020	Cross-sectional	238	15.64-17.99	COVID-19 pandemic	Online questionnaires, Home Adjustment to COVID-19 Scale, Adolescent Routines Questionnaire, COVID-19 Adolescent Symptom and Psychological Experience questionnaire	Descriptive statistics, independent samples t tests, regression analyses
Bobo et al. (2020) (13)	6 April 2020	Qualitative- case study	533	NR	Lockdown	Online questionnaire	Descriptive, qualitative and textometrical analyses
Bubb & Jones (2020) (22)	22 April-1 May 2020	Mixed design	1995	NR	Lockdown	Online questionnaire	Thematical analyses
Dong et al. (2020) (11)	March 2020	Mixed design	3275	20-50	COVID-19 pandemic	Online questionnaire, Demographic information, Online learning during COVID-19,Parents' beliefs and attitudes around online learning	Quantitative and qualitative approaches,

							descriptive statistical analysis
Goodrich et al. (2020) (14)	November 2020 and Fall 2020	Cross-sectional	9104	NR	COVID-19 pandemic	Online surveys	NR
Kuhfeld et al (2020) (43)	March-September 2020	Qualitative-projections	5 million	NR	COVID-19 pandemic	Projections answering four research questions	Projections on actual data
Maldonado & De Witte (2020) (35)	2015-2020	Mixed design	5832	NR	COVID-19 pandemic	Standardized tests that are administered every year by the network of catholic schools in Flanders, telephone surveys	T-tests, difference-in-differences (DiD) estimation
Moghli & Shuayb (2020) (15)	5 May 2020	Cross-sectional	678	NR	COVID-19 pandemic	Online questionnaire	Cross tabulation method
Putri et al (2020) (16)	NR	Case study	15	27-54	COVID-19 pandemic	Semi-structured questions through interview	Thematic data analysis guidelines
Sintema (2020) (48)	NR	Case study	3	NR	COVID-19 epidemic	Semi-structured interviews via telephone	Appropriate qualitative data analysis techniques
Tomasik et al. (2020) (23)	19 January 2020 and 11 May 2020	Cohort	28685	9-15	COVID-19 pandemic	MINDSTEPS assessments for mathematics and German	Multi group second-order piecewise latent growth model
Zhang Qing et al. (2020) (7)	10 February- 22 March 2020	Cross-sectional	896	12–14	COVID-19 pandemic	Emotional resilience scale-Chinese version, Middle school student learning skill evaluation questionnaire	T-testing, Pearson's correlation, and multivariate linear regression.
Zhao Ying et al. (2020) (8)	7-13 March 2020	Cross-sectional	2010	Students 8-13, parents 30-50, teachers 30-50	COVID-19 pandemic	Online questionnaire, Strengths and Difficulties Questionnaire (SDQ), Self-Rating Anxiety Scale (SAS)	chi-square test
Banerjee et al.(2021) (17)	1 July 2020 and 31 October 2020	Cross-sectional	53	NR	Lockdown	Online questionnaire	NR

Baschenis et al. (2021) (18)	2019 and March-June 2020 and August 2020	Cross-sectional	119	7-14	Lockdown	Online questionnaire, Battery for the Assessment of Developmental Dyslexia and Dysorthography-2, Assessment of Reading and Comprehension Skills for Elementary and Middle School	Descriptive statistics, t-test, $\chi 2$ tests
Catalano et al. (2021) (44)	April of 2020	Mixed design	300	NR	COVID-19 pandemic	Online questionnaire	NR
Clark et al. (2021) (37)	September 2017-January 2020	Cohort	1835	NR	Lockdown	Exam results in the five compulsory subjects (Chinese, Math, English, Politics, and History),	Difference-in-differences (DID) estimation
Cui et al (2021) (38)	NR	Cohort	1008	NR	COVID-19 pandemic	Online questionnaire	Student t test, chi-square tests or Fisher exact tests
Engzell et al (2021) (24)	2017-2020	Cohort	289189	8-11	COVID-19 pandemic	Biannual test scores in core subjects (math, spelling, and reading)	Difference-in-differences design
Gore et al (2021) (50)	2019-2020	Cohort	3030	Mean 9,7	COVID-19 pandemic	Progressive Achievement Tests (PATs) in mathematics, reading and science	Linear mixed models
Hernawati et al. (2021) (39)	February-March 2021	Qualitative description	29	NR	COVID-19 pandemic	Online survey	NR
Lichand et al. (2021) (45)	2018-2020	Cohort	8510720	NR	COVID-19 pandemic	Test scores	Differences-in-differences strategy, Ordinary Least Squares regressions (OLS)
Ma et al. (2021) (40)	11-17 April 2020	Cross-sectional	668	NR	COVID-19 pandemic	Online survey, Impact of Events Scale-Revised (IES-R), Short Mood and Feelings Questionnaire (SMFQ-P)	Multiple logistic regression analysis
Mælan et al (2021) (25)	October-November 2018 and 2020	Cross-sectional	6640	NR	COVID-19 pandemic	Online questionnaire	One-way analysis of variance (ANOVA)

Meeter (2021) (26)	2018-2019 and 2019-2020	Cross-sectional	100471	NR	Lockdown	Adaptive practicing software for teaching mathematics (Snappet)	Regression analyses, ANOVA
Patarapichayatham et al. (2021) (46)	2019-2020 and 2020-2021	Cohort	326453	NR	COVID-19 pandemic	Data from the extensive I station database	Piecewise growth model
Poulain et al (2021) (27)	2016-2020	Cohort	285	1-10	Lockdown	Data from LIFE Child study	Linear or proportional odds logistic regression analyses
Sabates et al. (2021) (49)	2016-2018	Cohort	1166	8-14	COVID-19 pandemic	Data from Complementary Basic Education (CBE) program and Early Grade Mathematics Assessment (EGMA).	Difference-in-difference (DID) estimation techniques
Scarpellini et al. (2021) (28)	8-15 May 2020	Cross-sectional	1601	34-49	COVID-19 pandemic	Online structured questionnaire	Chi-square or Fisher's exact test, Wilcoxon's test
Schult et al (2021) (29)	2015-2020	Cohort	999419	NR	COVID-19 pandemic	Large-scale assessment results in reading and mathematics from annual mandatory tests, "Lernstand 5"	Descriptive statistics, bivariate correlations
Sibley et al. (2021) (47)	2015-2018 and April–June 2020	Mixed design	412	13-22	COVID-19 pandemic	Long-term follow-up assessment consisting of parent and self-ratings of symptoms and impairments, either online or by phone	Multilevel models, Analysis of Variance (ANOVA)
Soriano-Ferrer et al. (2021) (19)	November 2019-January 2020 and May 2020	Cohort	32	9-14	Lockdown	Factor "g" intelligence test, Standardized reading skills battery, Children's depression inventory-short form, State-trait anxiety inventory for children, Motivation to read profile-revised, Task-value scale for children, Reading activity inventory	Descriptive statistics, t tests, multiple linear regression analyses
Spitzer & Musslick (2021) (30)	15 March-15 June 2019 and 15 March-15 June 2020	Cohort	13249	NR	COVID-19 pandemic	Data from the Bettermarks software for mathematics	Linear mixed model

Siachpazidou et al. (2021) (31)	27 November-3 December 2020	Cross-sectional	482	NR	COVID-19 pandemic	Online questionnaire	Two tailed Pearson's, chi-squared or Fisher's exact test, bivariate analyses
Steinmayr et al. (2021) (32)	April-July 2020	Cross-sectional	2647	Mean 43.37	COVID-19 pandemic	Online questionnaire	Descriptive statistics, structure equation models (SEMs)
Tus (2021) (41)	2020-2021	Cross-sectional	493	NR	COVID-19 pandemic	Online questionnaire, Parental Involvement Questionnaire	Statistical techniques such as frequency count, percentage, and mean
Vainikainen et al. (2021) (33)	April 2020	Cross-sectional	101160	NR	COVID-19 pandemic	Nationally representative survey data, online questionnaire	Multilevel structural equation modelling, t-tests
van der Velde et al (2020) (34)	2018-2019 and 2019-2020	Cross-sectional	133450	NR	Lockdown	Data from SlimStampen software	Regression models
Yayci et al (2021) (42)	NR	Qualitative- case study	74	NR	Lockdown	Online questionnaire	Descriptive analysis method
